# Norm-focused nudges influence pro-environmental choices and moderate post-choice emotional responses

**DOI:** 10.1371/journal.pone.0247519

**Published:** 2021-03-01

**Authors:** Carlos Andres Trujillo, Catalina Estrada-Mejia, Jose A. Rosa

**Affiliations:** 1 Universidad de los Andes–School of Management, Bogotá, Colombia; 2 Iowa State University–Debbie and Jerry Ivy College of Business, Ames, Iowa, United States of America; Middlesex University, UNITED KINGDOM

## Abstract

In this paper, we use choice architecture techniques to activate both social and personal norms, seeking to increase pro-environmental choices and to better understand the effect of such norm types on post-choice emotional responses. In four experiments, we make different social or personal norms salient by aligning choice environments with psychosocial mechanisms that activate different types of norms. We use different choice architecture techniques to change information, alter product sets, and generate the social consequences of choices. The target behavior, purchasing a recycled paper notebook, is captured through direct purchase behaviors or willingness to pay commitments. We find that choice architecture activates personal but not social norms, and that associated positive and negative emotions (guilt, shame, regret and pride) are elicited by choices but not by willingness to pay. Moreover, manipulating choice environment moderates the relationship between choice and norm-related emotions, such that positive emotional responses seem to be stronger than negative ones. The results suggest that choice architecture interventions can activate individual level beliefs about sustainability and help reduce the attitude-behavior gap.

## Introduction

Almost twenty years since Robbins [1] argued for more responsible consumption behavior, helping individuals make pro-environmental decisions remains a focal goal for decision-making scholars in multiple fields. However, behaviors are changing at a slower pace than the planet requires to stop climate change. We approach this challenge by accepting that many people have admirable intentions on which they fail to act, a phenomenon that has been referred to as the attitude-behavior gap [2, 3]. Given that consistent responsible consumption is difficult (e.g. [3]) in spite of its economic, social, and environmental benefits, our concern is to identify interventions that help bridge the attitude-behavior gap. We focus on situational interventions that nudge people along environmentally friendly paths by peripherally activating different types of norms. Our focus is to use choice architecture techniques (e.g., changing product descriptions, highlighted information) to make either social or personal norms salient, and study the effect of such manipulation on short term behaviors and post-choice emotional mechanisms that potentially favor the long-term adoption of pro-environmental preferences. We aim to show that nudging people to follow different types of norms can potentially activate such preferences by virtue of the integral emotional responses triggered by either following or not following the intended intervention. We posit that people who follow the activated norms will experience a positive emotional response that reinforces such behavior in future episodes, whereas people acting against the activated norms will experience a negative emotional response that also reinforces the target behavior.

Nudging–using choice architecture to influence behavior without forbidding options or significantly changing economic incentives [4, 5]–has gained popularity as a tool to promote socially desirable behaviors; but the emotional consequences of nudging and its promotion of pro-environmental behaviors (PEB) have not been generally explored. While this is a broad research gap, our study offers novel insights toward filling it by tapping specifically into the emotional experience of behaving consistently or inconsistently with activated norms. We use norms to test this potential effect of nudging because there is ample research on both their effect on PEB and the emotional responses that accompany compliance and violation of both social and personal norms. A rich body of evidence argues that social norms impact several PEB, including energy use, recycling, green consumption, littering, water conservation, and towel reuse (see [6] for a recent meta-analysis on field interventions). However, recent works counter argue that social norm effects are not always as strong as some of the original work suggests. For example, studies have found that normative messages were not more effective than the standard message to promote hotel guests’ towel reuse [7, 8 Study 1]. In a similar vein, Nisa, Varum and Botelho [9] showed that the effect of social norms in promoting towel reuse was weaker than what was reported in previous studies. Additionally, a recent review shows that social norms were ineffective for a variety of other PEB [10, Table 1]. Evidence for the influence of social and personal norms is not equally abundant. Although the literature also reveals a relationship between personal norms and PEB [11, 12], the evidence on the influence of personal norms on PEB is scarce.

**Table 1:** Norms description.

Type of norm		Description	What motivates individuals to conform to the norm	How deeply that norm has been internalized
Descriptive norms		Indicate what most people do.	Action is likely to be effective or adaptive, and/or helps coordinate behavior	Does not need to be internalized
Injunctive norms	Subjective social norms	Indicate what most people think the individual should do.	Enforced by external rewards and/or punishments	Does not need to be internalized
	Personal introjected norms	Indicate what the individual should do, with pressure coming from internally administered rewards.	Enforced by feelings of guilt, pride or ego enhancements	Superficially internalized
	Personal integrated norm	Indicate what the individual should do through a well-crafted rationale grounded on personal values and goals.	Enforced by feelings of consistency with internalized values and norms	Deeply internalized

Note. This table is based on the descriptions proposed by Thøgersen [21].

Given the attention that social norm interventions have garnered as policy tools to promote PEB, it is crucial to better understand how to activate social and personal norms to influence PEB. This research contributes to partially fill those research gaps, by specifically studying the effectiveness of nudging on both types of norms. Moreover, in looking for ways to reduce the attitude-behavior gap in the midst of an over saturated flow of communications to consumers, we particularly test whether the activation of personal norms through the choice environment may be more effective than appeals to social norms.

Summarizing, we use four experiments to make social and personal norms salient through choice architectures [4, 5, 13]. The research unfolds in two steps. First, we explore differences in how norm types elicit pro-environmental product choices. Second, we explicitly compare the effectiveness of personal and social norms in shaping pro-environmental preferences, and the ability of manipulated choice environments to moderate the relationship between expressed preferences and elicited emotions that potentially engage feedback learning systems. To put our contributions in perspective, we review the literature on social and personal norms vis a vis PEB, the relationship between norms and discrete feelings, and choice architecture intervention typologies.

## Theoretical background

### Social and personal norms

Norms are behavioral guidelines for many activities, including consumption. They may indicate required actions, or identify permissible and forbidden behaviors for specific situations [14, 15]. Norms come in many variants and multiple taxonomies have been proposed (e.g., [16–20]). Thøgersen [21] classified norms based on individual motivations and how deeply the norms have been internalized. Based on these attributes, norms are divided into descriptive (stating what is normally done) and injunctive (stating what is approved or disapproved). Descriptive norms provide information about “what most people do” and what actions are most likely effective and adaptive in different situations [18]. It has been suggested that people comply with descriptive norms (i.e., imitate others) because the norms contain useful information about adaptive behaviors (e.g. [18]) or because they allow people to coordinate their actions effectively [18]. Table 1 further describes these categories.

Injunctive norms, instead, indicate how one should act in certain situations. They serve as standards or guidelines for appropriate behaviors [17, 18]. Injunctive norms can be further divided into subjective social norms and personal norms. For a norm to be subjective social, the individual must believe that others have opinions about what constitutes correct behavior in certain situations [15–17]; and that those others will punish or stigmatize violators [22, 23]. At the heart of subjective social norms is the idea that people adhere to such norms because of social pressures and threats of punishment. Since these social norms are externally enforced through rewards and punishments, they do not need to be internalized to regulate behavior [15].

In contrast, personal norms inform individual behaviors that are not contingent on what others are doing, but depend instead on personal considerations or beliefs about right and wrong. People comply with personal norms even when outside pressure does not exist; and may even go against social pressure [16, 17, 24]. Personal norms can be differentiated further into introjected and integrated norms [21]. Introjected norms are superficially internalized personal norms. That is, the individual has adopted the behaviors to a degree, but without reflecting on how the underlying norms relate to personal values and goals [21, 25, 26]. Compliance with introjected norms is reinforced by self-administered rewards and punishments (e.g., enhanced self-esteem, pride, guilt, shame), not from the environment [21]. Social pressure is replaced by internal pressure [27, 28].

A greater degree of internalization occurs when the individual has reflected on how norms and compliance relate to personal values and goals [21]. Integrated personal norms are partly or wholly enmeshed with the individual’s self-concept, and grounded on personal values and goals [21, 25, 26]. Integrated personal norms do not need reinforcement from guilt, pride or ego enhancements, but they can give rise to moral emotions and self-regulation [29].

Descriptive, subjective social, and personal (integrated and introjected) norms have been invoked to explain environmentally responsible behavior (e.g., [30–32]). For instance, descriptive norms (i.e., telling people what others did) were effective in reducing towel use in hotels [33], reducing energy use [34] and increasing recycling [35]. Moreover, a recent development of how descriptive norms are expressed introduced the notion of dynamic vs. static descriptive norms [36]. The dynamic form articulates that others are “increasingly” adopting a target behavior. This form has been shown to be effective in promoting PEB such as reducing meat consumption and avoiding disposable to-go cups in cafes [37]. Subjective social norms (i.e., telling people what others expect them to do) have been effective in reducing waste [38] and residential water usage [39]. Finally, personal norms (e.g., duty to society) have effectively triggered behaviors such as reducing environmental theft [40] and increasing environmental cause donations (e.g., [12]).

### Norms and norm-related emotions

One mechanism by which norms influence behavior is by activating norm-related emotions [16]. Social norm transgressions typically generate shame when the transgressions are observed and judged by others while personal norm violations typically generate guilt over wrongdoing [41, 42]. We, however, consider that the emotional activation triggered by personal norm violations can be enriched when considering the internalization levels. Guilt, shame and regret are linked to moral self-regulation (for a review, see [29]) which engender emotional and behavioral responses that tap personal norms internalization levels. Guilt typically triggers restitution because it is activated by the transgression. Shame focuses more on the self-evaluation than on the action. As such, it can be expected that behaviors that violate low-level internalization norms (i.e., introjected personal norms) will elicit guilt but not shame, whereas behaviors that violate highly internalized norms (i.e., integrated personal norms) should elicit both guilt and shame. Moreover, internalization may also influence the elicitation of regret in connection to guilt. For integrated personal norms that tap into moral regulation, guilt is accompanied by a negative feeling of regret for violating internalized standards, which motivates restitution and even self-punishment (see [29]). Hence, guilt that is not accompanied by shame and regret, denotes low internalization of personal norms.

Conversely, abiding by personal norms is associated with pride [43]. Again, we propose that considering the internalization levels enriches the comprehension of the emotional activation triggered by personal norm compliance. Acting consistently with a low-level internalization of norms (i.e., introjected personal norms) will elicit pride for doing what is right, expected or correct [21]. Moreover, complying with highly internalized norms (i.e., integrated personal norms) may foster a positive self-concept, and thereby elicit not only a positive feeling of pride but also a sense of being a good person. Table 2 summarizes our expected emotional responses.

**Table 2:** Expected emotions by condition.

Type of norm	Salient Emotional responses
Descriptive social norm	Shame
Introjected personal norm	Guilt and Pride
Integrated personal norm	Guilt, Shame, Regret, Pride and Feeling as a good person
Subjective social norm	Shame

These emotions and the ensuing behavioral tendencies are relevant for PEB. Previous research has established the low link between guilt [44], pride [45, 46], shame [47], and sustainable purchase behavior. Additionally, studies have shown a positive relationship between guilt and pro-environmental actions such as recycling [48], use of public transportation [49], and support for climate change policy [50]. Similarly, there is evidence for a positive association between experiencing shame and PEB [47]. On the other hand, recent studies have identified a positive relationship between pride and intending to buy environmentally friendly products [45, 51]. Finally, acting environmentally-friendly had lead people to feel like a good person [52 Study 2, 53].

To explain how these emotions affect behavior, we use the perspective of the theory of feelings-as-information [54], which argues that people attend to their feelings as a source of information that is useful in judgements and decisions. More specifically, people threatened by environmental deterioration will experience negative emotions (i.e., shame or guilt), and their pro-environmental reactions will be aimed at relieving such negative feelings. Alternatively, people praised for preventing or slowing down environmental degradation will likely experience positive feelings and appraisals of those feelings (i.e., pride or sense of being a good person); PEB will be aimed at maintaining such positive feelings. This way, the individual learns the association between PEB and specific emotions, which may reinforce the formation of pro-environmental preferences in the long term.

### Choice architecture

Choice architecture [4, 5] focuses on the way that choice presentation influences decisions, which is foundational to nudging initiatives. Choice architects can influence decisions in many ways, such as by varying the presentation order of alternatives, how attribute information is provided, and by defaults selection. An expanding body of research brings to light the positive effects that choice architecture can have on real world decisions [4, 5, 55, 56]. Münscher, Vetter and Scheurle [13] present a review and taxonomy of available choice architecture techniques. They divide the techniques into three major groups. The first group of architecture techniques involve directly assisting decision makers through reminders and personal or public commitment. The positive effects of making public commitments have been found in studies on recycling behavior [57, 58].

A second group includes techniques that target the presentation of decision-relevant information without altering the options. A well-known example of this type of interventions is reframing the information; that is, presenting the same (equivalent) information in different ways [59–61]. For example, it has been demonstrated that negatively framed messages (i.e., highlighting the harmful environmental consequences of buying unsustainable alternatives) are more effective than positively framed ones (i.e., highlighting the environmental benefits of green options) when encouraging pro-environmental purchases (e.g. [62, 63]). Another example of this type of interventions is to provide additional information about how others behave in similar circumstances, and to inform buyers about germane descriptive social and personal norms. For instance, individuals informed about aggregated energy consumption or their neighbor’s recycling behavior have at times adjusted their own behaviors [64–66]. Moreover, dynamic expressions about others’ use of non-disposable mugs have led to reduced use of to-go cups in cafes [37].

The third group involves managing the decision structure by modifying the available options in the decision situation, including their range or composition, changing the default option, or adjusting the effort required for selecting an option and the consequences of selecting it. Choice architects, for example, can introduce a decoy alternative (an asymmetrically dominated option) to a choice set to influence consumer decisions. The decoy alternative is dominated by one item in the set but not by another. Adding such an alternative to a choice set has been shown to increase the probability of choosing the item that dominates it [67, 68]. Choice architects can also modify the consequences of decision options, such as when potential impact to a buyer’s social integrity is mentioned as a consequence beyond monetary costs and benefits. In a study by Griskevicius, Tybur and van der Bergh [69], activating status as a motive made participants choose environmentally friendly products more often in a situation where positive self-presentation through choice–behavior was possible. Our study uses three different techniques (i.e., we manipulate decision-relevant information about the products, manipulate the decision structure by varying the available product set at the time of purchase, and manipulate the social consequences of buyer decisions by making their choices public) with the specific goal of highlighting different types of social and personal norms. We elaborate more on the specific details further in the methods section.

### Summary of research goals and hypotheses

This study focuses on using choice architecture to activate descriptive social norms in some cases, and personal norms in other cases, in order to promote pro-environmental purchase preferences that are elicited either as direct choices or value estimations. We expect that decision environments that activate social or personal norms will induce pro-environmental choices more frequently and encourage higher product value estimations than non-norm decision environments. We also test the moderating effect of the choice architecture interventions on levels of norm-related post-decision negative and positive emotions. In particular, we expect that the activation of personal norms should be related to guilt and pride, whereas the activation of social norms should be related to shame. Similarly, the activation of non-internalized personal norms should elicit guilt and pride whereas the activation of internalized norms should also elicit guilt accompanied by regret, shame, pride, and a sense of being a good person (see Table 2 as a reference for the type of norm and emotions response).

Assessing the elicitation of such discrete emotions and the moderation effect of choice environments is relevant for two reasons: First, based on extant knowledge about the link between norm type and emotions, the elicitation of specific feelings corroborates the activation of targeted norm types. In effect capturing discrete emotions triggered by the experimental tasks serves as a manipulation check. Second, emotion elicitation activates learning. Theories on emotions as factors in decision feedback systems (e.g., [70]) argue that people learn from the emotional consequences of decisions through cognitive processing that associates emotional states and behaviors. During decision making, anticipation [71] and activation of learned emotions plays a key role in driving preferences, as people seek or avoid learned emotional outcomes (e.g., avoid regret, seek pride). Although we do not directly test learning, this research explores the possibility of stimulating the activation of such emotions through choice architecture as part of interventions, which may favor the long-term learning of pro-environmental consumption preferences.

## Methods

### Overview of the experiments

In four experiments, we use actual purchasing choice or willingness to pay for an environmentally friendly notebook as a preference elicitation method. Participants’ decisions are implemented by the actual acquisition of the notebook. We contracted with a notebook manufacturer to produce environmental-friendly paper and regular paper samples, to ensure they were indistinguishable in all other aspects except price and to have a notebook that was not available in the regular market. The study uses different choice architecture techniques [13] to activate social and personal norms. In Experiments 1A and 1B, descriptive social norms and introjected personal norms are made salient by changing decision information about products. Experiment 2 makes integrated personal norms salient by manipulating the buyer’s choice set. Experiment 3 appeals to subjective social norms and indirectly makes them salient by manipulating post-decision social consequences and public exposure. After each experiment, we tested the moderating effect of the choice architecture interventions on levels of norm-related post-decision negative and positive emotions (i.e., guilt, shame, pride, and sense of being a good person).

All the experiments follow a similar procedure. Here we explain the general experimental flow and later in the paper we describe variations. The experiments were conducted in a closed lab setting and programmed in Qualtrics. Informed consent was obtained from all participants at the beginning of each session. The informed consent appeared in the first screen of the experiment, in which subjects were informed of the general procedure and implications of their voluntary participation. Following this explanation, they had to agree to participate before continuing. The ethics committee of Universidad de los Andes, School of Management, where the experiments took place, approved the project. The university provided project funding. Participants for all the experiments were recruited through public announcements in mailing lists of several schools across the university. The advertising mentioned participants would be asked to make consumption decisions and paid based on those decisions. All experiments were planned as part of one study design and conducted within the same week. To ensure that the respondents participated in only one of the experiments, we asked them if they had already participated before entering the lab, and double-checked their identification document.

At the start of the session, participants completed 10 simple addition problems (e.g., 10+2*9) and received $0.70 credit for each correct answer, to a maximum credit of $7. We asked them to “work” for money they could spend later. We implemented this procedure to make sure they had extra money to spend, reducing heterogeneity in participant discretionary income levels, and to induce ownership. Next, they were told they would have access to a virtual store that had a number of products from which they could choose, that they had to buy one product using their earnings, and that for 20% of participants the decision would be enforced. The University did not have an official virtual store at that time, and therefore, the store was presented as a prototype of the Internet-based university store. After reviewing the information, participants were asked to either choose one product (Experiment 1A, Experiment 2) or give their willingness to pay (Experiment 1B and Experiment 3) for one of the two notebook options. Participants were then asked to report the emotions felt toward their decision.

Finally, participants completed the following questions that served as covariates for all analyses. They answered two questions to verify how often they bought eco-friendly notebooks and their familiarity with similar notebook prices. Then, they completed dispositional measures of concern for the environment (adapted from [72]), the Attention to Social Comparison scale [73] and provided demographic variables. Covariates were included to limit potential confounds. For example, the measure of concern for the environment captures ex-ante heterogeneity in environmental concerns that may increase preferences for environmentally friendly products. The attention to social comparison measure helps control for heterogeneous sensitivity to the opinions of others. All measures are described below and descriptive statistics are reported in Table 3.

**Table 3:** Descriptive statistics for all experiments.

	Experiment 1A		Experiment 1B		Experiment 2		Experiment 3	
	Mean (SD)	Alpha	Mean (SD)	Alpha	Mean (SD)	Alpha	Mean (SD)	Alpha
Guilt	1.71 (1.15)		1.77 (1.24)		1.63 (1.14)		1.53 (.93)	
Shame	1.40 (.80)		1.54 (.98)		1.65 (1.20)		1.65 (1.07)	
Regret	1.74 (1.06)		1.57 (1.01)		2.00 (1.53)		1.55 (.96)	
Pride	3.84 (1.52)		3.88 (1.48)		3.75 (1.75)		3.93 (1.41)	
Sense of being a good person					3.84 (1.68)		4.33 (1.22)	
Attention to social comparison	3.60 (.73)	0.87	3.44 (.76)	0.76	3.67 (.79)	0.82	3.44 (.92)	0.87
Concern for the environment	4.08 (1.03)	0.78	4.23 (1.08)	0.81	4.26 (1.17)	0.88	4.40 (.88)	0.77
Familiarity notebooks prices	2.56 (1.44)		2.16 (1.26)		2.39 (1.42)		2.55 (1.57)	
Purchase frequency	2.91 (1.34)		2.96 (1.38)		3.16 (1.52)		2.90 (1.43)	

Note. All variables were measured with a 1 to 6 scale. Cronbach´s Alpha is reported.

#### Emotions

We included one positive (pride) and three negative (shame, guilt and regret) emotions in Experiment 1A and 1B. We added a meta-cognitive affective evaluation of “feeling as a good person” for Experiment 2 and 3. We asked participants the question “How do you feel about the decision you just made” followed by four statements of the form “I feel guilty” “I feel proud” and so forth. All emotions were measured on a 6-point scale (1 = completely disagree, 6 = completely agree). This procedure helps to activate affect-as-information mechanisms by directing participants to a) acknowledge specific feelings and 2) link them to the decision or judgment just made.

#### Concern for the environment

We measured concern for the environment with a 5-item scale adapted from [72] (for example, “I see myself as a person concerned about environmental problems”). Items were measured on a 6-point scale going from 1 (Doesn’t describe me at all) to 6 (Describes me very well).

#### Attention to social comparison

We measured the extent of participants’ sensitivity to social comparison using the 12-item scale developed by [73] (for example, “When I am uncertain how to act in a social situation, I look to the behavior of others for cues”). Items were measured on a 6-point scale going from 1 (Always false) to 6 (Always true).

#### Eco-friendly notebooks purchase frequency and familiarity with notebooks prices

Study participants indicated how often they bought eco-friendly notebooks on a 6-point scale (1 = never, 6 = very frequently), and their familiarity with similar notebook prices on a 6-point scale (1 = I do not know the prices of this type of notebook, 6 = I know very well the prices of this type of notebooks). Finally, they indicated their age and gender.

#### Participants’ payment was determined as follows

Before the start of the experiment participants were informed that for 20% of the participants the decision would be enforced, and therefore, they would have to buy the notebook they had chosen. To select the participants whose decisions were binding at the end of the experiment, participants were asked to draw a ball from a bag with five balls, four blue and one pink. For those who drew a pink ball the decision was enforced (they bought the notebook) using their earnings. When participant decisions were enforced, final payments were $7 minus the selected product’s price and the purchased notebook (Experiment 1A, Experiment 2, and Experiment 3). In Experiment 1B the payment was $7 minus the amount they offered for the product plus the product.

When decisions were not binding (80% of cases), payments were decided by a lottery that paid $1.8, $3.5 or $5 with equal probability. These procedures, explained before the start of the experiment, ensure that participants did not perceive the bindings scenario as disadvantageous or had a way of strategic playing to win a higher payment. Hence, participants had no incentive to not act according to their true preferences when choosing the notebook.

### Plan of analysis

The data will be analyzed in three phases. First, descriptive contrasts are used. In the experiments where choices are elicited, we compare the relative frequencies of buying the two types of notebooks. In the experiments where Willingness to Pay (WTP) is captured, we report the mean values. Second, for both choice and WTP experiments, we assess the influence of the treatment conditions using treatment effects analysis, in which we estimate the Average Treatment Effect of the Treated participants (ATET). This technique is based on the estimation of the effect size of the treatment conditions on the desired dependent variable using the average of the individual differences rather than the difference in means of the different groups [74]. Third, we look at the effects of Choice and WTP answers on emotions and the moderating effect of treatment conditions on such relationships. In order to do so, we start by reporting the main effects of Choice/WTP on every measured emotions using standard OLS regression models. Then, to establish the moderating effect of treatment conditions, we use the procedures recommended by [75] using the specially designed PROCESS plug-in for SPSS. In this procedure, after conducting linear regressions that include the interaction terms of Choice/WTP with dummy variables for treatments, the moderations are probed by the post regression estimation of the effect size of Choice/WTP on every emotion for each experimental group as defined by the treatment dummies. This is known as “Contingent Analysis” because it provides the estimated effects of interest given certain values of the moderator. In our case, since our moderation variable is categorical, we report an estimation of the effect of Choice/WTO on emotions for each treatment.

#### Experiment 1A

*Methods*. *This experiment involved choice architecture interventions in w*hich product information is manipulated to influence product choice. Either introjected personal norms or descriptive social norms were made salient in the different conditions before participants had to choose between an eco-friendly paper notebook and a regular paper notebook. Self-assessed emotion data were gathered after choices were made.

*Participants*. To conduct a priori power analysis, we assessed the effect of treatment and other covariates on the percentage of people that chose the eco-friendly notebook, using a logistic model. We used G*power software, following the procedures described by Faul, Erdfelder, Buchner and Lang [76] for z-test of regression coefficients in logistic models. To detect an effect size of 15% over the null hypothesis of random choice (50%) (i.e., odds ratio = 1.85) with alpha = 0.05 and a power of 0.8, the resulting target sample size was 77 with critical z = 1.64. We increased the sample size above the recommended number to observe effects smaller than 15% with sufficient statistical power. There is no clear antecedent about the expected effect size, so we targeted an effect with meaningful economic interpretation, but we increase the sample sizes in order to detect smaller effects. In total, 139 undergraduate students participated in this experiment (*M*_*age*_ = 20.4, *SD* = 2.4; 43.2% female), no participant was excluded.

*Procedure*. Participants were randomly assigned to either a descriptive social-norm condition, an introjected personal-norm condition, or a control no-norm condition. After completing the math task described in the overview, participants entered the virtual store and were presented with information on two products–an eco-friendly paper notebook and a regular paper notebook. The information consisted of a photograph and five information bullet-points. Notebook dimensions, number of pages, product labels and prices (i.e., eco-friendly paper notebook = $5.50; regular paper notebook = $4.20) were held constant. Prices were determined using average market prices of notebooks. In the descriptive social-norm condition we used an explicit reference to the norm (See [6]) where the eco-friendly notebook was described as using recycled paper and participants were told that *a large number of students use such ecological products*. For the introjected personal norm condition, we used the same description for the eco-friendly notebook and told participants that *choosing the ecological notebook is the right thing to do*. Explicit priming of the correct, morally charged behavior, is used to activate superficially internalized personal norms (i.e., introjected). In the no-norm control condition, participants were provided with only the description of the recycled paper. In all conditions, the regular paper notebook description detailed the type of regular paper (see Appendix A for full description of stimuli). Study participants chose the product by clicking on an image. Next, they reported their emotions and the covariate measures explained in the experiments’ overview.

*Results*. In the introjected personal-norm condition 61% chose the eco-friendly notebook and 39% the regular notebook (*z* = 2.88, *p* < .003). In the descriptive social-norm condition, the choice proportion was exactly 50% and 50% (*z* = 0, *p* = 1) and in the no-norm control condition the proportions were 53% and 47% (*z* = .91, *p* = .35). Additional descriptive statistics for all experiments are shown in Table 3. To estimate the effect size of the intervention on the probability of choosing the eco-friendly notebook, we conducted a treatment effects analysis (with probit model, regression adjustment estimation, and robust standard errors), controlling for emotions (guilt, regret, shame, and pride), and pro-environmental and social comparison measures. We control for emotions because of mechanisms of anticipation explained earlier. The self-reported emotions that we captured are unlikely to be a state strictly elicited after the experimental task. Rather, it may be a retrospective account of the emotions experienced throughout the decision-making process.

The analysis revealed a significant effect of introjected personal-norm treatment on the probability of choosing the eco-friendly notebook (Average Treatment Effect of the Treated (ATET) = .16.9 (16.9%), *s*.*e*. = .08, *z* = 1.94, *p* = .05) over the descriptive social-norm highlighting, which in turn had not an statistically significant effect on buying the eco-friendly notebook when compared to the control treatment. (ATET = .09 (9%), *s*.*e*. = .09, *z* = 1.01, *p* = .31). This indicates a 16.9% increase in the probability of choosing the eco-friendly notebook when introjected personal norms are made salient when compared to the descriptive social-norm treatment.

We then tested the effect of choosing the eco-friendly notebook on each emotion using standard OLS linear regressions to estimate the main effects of choice on self-reported emotions before assessing treatment moderation, using the same covariate variables as in the treatment effects analysis. We captured emotions once the experimental task was completed and we asked participants to report their emotions towards the task. As expected, we found that choosing the eco-friendly notebook reduces guilt (B = -1.13; t = -4.18; p = .00), reduces shame (B = -.60, -3.23, p = .00), and to a lesser extent, regret (B *=* -.49; t = -1.93; p = .05). It also increases pride (B = .87: t = 2.57; p = .01). To test if norm-activation moderates the relationship between choice and emotions, we conducted conditional regression analysis, as previously explained [75], to check the type of norm that was activated based on theory-grounded projections of the relationship between norms and emotions. As stated, we expected personal-introjected and descriptive social norms to strengthen the link between choice and inward- (guilt/pride) or outward- (shame) looking emotions respectively. We proceeded with the moderation analysis by setting self-reported emotional states as dependent variables and the experimental treatments as moderators, including the no-norm control treatment. That is, we used a three-category moderator. We also controlled for the same covariates. Using the PROCESS macro [75], v 2.16 in SPSS with bootstrapped C.I (5000 repetitions) and mean-centered products, we estimated one regression per emotion (i.e., guilt, shame, regret and pride) including the interaction terms of treatment conditions with choice. Through these procedures, following extant knowledge on contingent effects assessed through regression [75], coupled with our experimental design, we were able to use choice behavior as the independent variable to elicit post-choice emotional states, whose effects are contingent on the treatment under which participants were randomly assigned. We report the contingent effect for each condition. In addition, we checked that the relationship between treatment and choice would not produce multicollinearity, and found that Variance Inflation Factors were all below 1.2. The results of the contingent effects estimations are in the bottom part of Table 4. Based on the estimated coefficients, the moderating effects are plotted in S1 Fig.

**Table 4:** Conditional linear regressions of chosen product on emotions, moderated by treatment.

	Model 1: Guilt			Model 2: Shame			Model 3: Regret			Model 4: Pride		
	Coeff. (s.e.)	t	p	Coeff. (s.e.)	t	p	Coeff. (s.e.)	t	p	Coeff. (s.e.)	t	p
Constant	1.29(.67)	-1.93	.06	1.26 (.49)	2.54	.01	1.44(.69)	2.09	.08	3.31(.92)	3.59	.00
**CHOICE (1 = ecofriendly)**	-1.05 (.33)	-3.16	.00	-.82 (.25)	-3.33	.00	-.56(.34))	-1.64	.10	.46(.46)	1.00	.32
Personal norm treatment dummy	.05 (.23)	.22	.83	-.10(.17)	-.60	.55	.06(.24)	.26	.80	.39(.32)	1.23	.22
Control treatment dummy	-.09 (.21)	-.40	.69	.00(.16)	.02	.98	.21(.22)	.98	.33	.00(.29)	.00	.99
Choice*Personal norm treatment	-.30 (.46)	.11	.91	.39(.34)	1.15	.25	.17(.47)	.36	.72	.65(.63)	1.02	.31
Choice*Control treatment	-.31 (.71)	-.65	.51	.22(.31)	.72	.47	.35(.43)	.80	.42	.98(.59)	1.69	.09
Attention to social comparison	.23 (.12)	1.92	.06	.09(.09)	1.02	.31	.20(.13)	1.57	.12	-.10(.17)	-.58	.56
Pro—Environmental predisposition	-.05 (.09)	-.52	.60	-.01(.07)	-.14	.89	-.11(.10)	-1.16	.25	.18(.13)	1.41	.16
Gender (1 = M)	-.33(.18)	-1.85	.07	-.21(.13)	-1.62	.11	-.08(.18)	-.45	.65	.04(.25)	.17	.87
Change in R2 due to interactions	.01	F = .44	.64	.01	F = -67	.51	.00	F = .32	.72	.02	F = 1.44	.24
Conditional effects of choice on emotions:												
Descriptive Social norm treatment	-1.05 (.33)	-3.16	.00	-.82 (.25)	-3.33	.00	-.56(.34)	-1.64	.10	.46(.46)	1.00	.32
Introjected Personal norm treatment	-1.35 (.34)	-4.01	.00	-.43 (.25)	-1.71	.09	-.39(.35)	1.13	.26	1.10(.46)	2.38	.02
Control treatment	-.96 (.28)	-3.45	.00	-.60 (.20)	-2.90	.00	-.22(.28)	-.76	.45	1.44(.38)	3.77	.00
	R2 = .27	F = 5.92	.00	R2 = .17	F = 3.34	.00	R2 = .08	F = 1.46	.18	R2 = .20	F = 3.98	.00
	N = 139			N = 139			N = 139			N = 139		

We found that for guilt, the type of norm induction (social or personal) had a small moderation effect; when probing the interaction, guilt was found to be negatively associated to choosing the environmentally friendly notebook under the social norm condition (B *= -1*.*05; t = -3*.*16; p =* .*00)*, but the effect size was 30% higher in the personal norm condition (B *= -1*.*35; t = -4*.*01; p =* .*00)*). In contrast, the relationship of pride and choice was moderated more strongly by norm activation. We found it to be positive under the personal norm treatment (B *= 1*.*10; t = 2*.*38; p =* .*02)* but insignificant under the social norm treatment. Looking at the socially related emotions, the relationship between shame and choice was moderated by norm-condition in the expected direction. In the descriptive social-norm treatment, the conditional effect of choosing the eco-friendly notebook on shame was negative (B *= -*.*82; t -3*.*33; p =* .*00)* whereas under the personal-norm condition the effect was not significant. As expected, regret was found to have no relationship with the chosen product. In the upper part of Table 4, we report the regression coefficients of the base regression with the interacting variables, their product and covariates. Note that the regression coefficients of the interacting variables represent simple, as opposed to main effects. This means that they should be interpreted as the effect of the variable when the other interacting variable is zero. In the bottom part of the table, contingent effects of Choice on emotions are estimated for each of the three experimental groups.

In sum, we found that activating personal-norms (versus descriptive social-norms) through information about behavior is more effective toward increasing buyers’ preference for the environmentally friendly notebook (16.9%) We also found that norm activation moderates the effect of choice on norm-related emotions.

#### Experiment 1B

*Methods*. This study tested whether the findings from Experiment 1A replicate when participants indicate their preferences by how much they are willing to pay (WTP) for an eco-friendly paper notebook instead of choosing between eco-friendly and regular paper options. In addition to capturing a different behavioral measure, WTP made it possible to rule out preference heterogeneity over prices for sustainability as an alternative explanation of pro-environmental choices in Experiment 1A. Such heterogeneity would be expressed through a trade-off between a product with superior sustainability (and higher price) and a product with average sustainability (and lower price but not sufficiently low to shift preferences to the non-eco-friendly notebook).

*Participant*. To conduct a priori power analysis, we evaluated the effect of treatment and other covariates on willingness to pay using a linear model. We used G*power software, and followed the procedures indicated by [76] for the deviation of a subset of linear regression coefficients from zero. Thus, to identify a small effect size based on Cohens f, we set (f^2^ = 0.1) with six covariates, alpha = 0.05 and power = 0.8. The resulting target sample size is 64 and critical t = 1.67. Once again, we chose to collect a bigger sample to increase the statistical robustness of subsequent analyses. The study involved 130 undergraduate students (*M*_*age*_ = 20.5, *SD* = 3.1; 38.5% female). No participant was excluded.

*Procedure*. Participants were randomly assigned to social-norm, introjected personal-norm or control conditions. Participants completed the math problems for monetary credit as explained earlier and accessed the virtual store. Once they entered the store, they were asked to indicate how much they were willing to pay for the eco-friendly notebook. All participants were then presented with information about the eco-friendly paper notebook. No information describing a regular paper notebook was presented. As in Experiment 1A, the information consisted of a photograph and four information bullet-point. Descriptions of the eco-friendly paper notebook in the social-norm, introjected personal-norm and the control conditions were the same as in Experiment 1A. After participants indicated willingness-to-pay (WTP), they reported on the emotions, completed the dispositional measures and answered the demographic questions explained earlier (see descriptive statistics in Table 3).

*Results*. Average WTP for the eco-friendly notebook was $2.92 (*SD* = $1.7) in the introjected personal-norm condition, $2.32 (*SD* = $1.5) in the social-norm condition, and $2.35 (*SD* = 1.6) in the control condition. Treatment effects analysis (linear model, regression adjustment estimation, robust standard errors) controlling for emotions (guilt, shame, regret and pride) and covariate variables showed a significant effect from introjected personal norms (ATET = .68 ($), *s*.*e*. = .33, *z* = 2.02, *p* = .04) over the control treatment and a non-significant effect from social norms (ATET = .16 ($), *s*.*e*. = .33, *z* = .49, *p* = .62) over the control treatment. People on average offered $0.67 more (approximately 30%) for the eco-friendly notebook when introjected personal norms were made salient.

We conducted standard linear regressions to analyze the main effect of WTP on emotions and to check that the relationship between WTP and treatment would not cause multicollinearity issues. Variance Inflation Factors were all below 1.2. We found that there was no significant relationship between WTP and emotions. Nonetheless, the intensity of emotions was not significantly different than that of Experiment 1A (see Table 3). This may indicate that choice and WTP elicit different psychological mechanisms. We further elaborate on this result in the Discussion section. Given that we found no main effects of WTP on emotions, analyzing a moderation effect of treatment on the relationship between WTP and emotions would not be meaningful.

In sum, results from Experiment 1B confirm that making introjected personal norms salient is more effective than making descriptive social norms salient to increase monetary offers for the eco-friendly notebook, as we reported above using treatment effect analysis. It appears, however, that the underlying computational mechanism that links introjected personal norms with WTP seems to neutralize or overshadow norm-related emotions. In addition, Experiment 1B confirms that given the average WTP for the notebook, the effects of Experiment 1A can be attributable to manipulation and not to the heterogeneity in the valuation of sustainability in the notebook.

#### Experiment 2

*Methods*. Experiments 1A and 1B showed that choice architecture could influence consumer preferences towards pro-environmental products more by directly making introjected personal norms salient through information manipulation than by making descriptive social norms salient trough similar information manipulations. In Experiment 2, we focus instead on highly internalized (integral) personal-norm activation. We test a different choice architecture by manipulating product assortment to test whether pro-environmental product choices are more likely either when the product is embedded in a strong pro-environment product set (such as would be found in the organic product or environmentally safe product sections in stores) or when the product set is pro-environmentally weak (e.g., environmentally safe products are distributed throughout the store). This manipulation appeals to integral personal norms because it can subconsciously activate the drive to buy the pro-environmental notebook through the implicit abundance/scarcity of responsible products. In our treatment, the scarcity of pro-environmental products indicates an undesirable consequence that triggers ascription of responsibility. This intervention implies that internalized personal norms may be activated by peripheral cues, even if decision makers are not fully aware of the action triggers in play. We expect participants to act on the situation (i.e., follow integral personal norms) such that the drive to choose the pro-environmental option will be higher under the environmentally weak product set. In contrast, in the environmentally strong set, participants may tacitly imply that the norm is followed and therefore that they do not need to act on it (i.e., weaker ascription of responsibility), reducing the drive to buy the pro-environmental notebook. Regarding emotions, we expect to observe the activation of guilt, shame, regret, pride and we introduce a “sense of being a good person” as an additional positive affective state that arises from self-evaluation of behaviors consistent with highly internalized norms (see [29]). If our hypotheses are correct, the role of emotions should be more salient in the weak set treatment than in the strong set treatment.

*Participants*. To conduct a priori power analysis, we again assessed the effect of treatment and other covariates on the percentage of people that choose the eco-friendly notebook using a logistic model. We set up the same parameters of Experiment 1A; that is, in order to detect an effect of 15% (Odds ratio = 1.85) over the null hypothesis of random choice (50%), with alpha = 0.05 and a power of 0.8, the resulting target sample size is 77 with critical z = 1.64. Experiment 2 participants were 80 undergraduate students (*M*_*age*_ = 20.1 (SD = 2.23; 48.1% female). No participant was excluded from the analysis.

*Procedures*. Participants were randomly assigned to one of two conditions: strong-environmental-product set vs. weak-environmental-product set. They completed the math task and were told the same cover story (i.e., university web store), but they were asked to first browse the store catalog, and look at one product at a time. Next, they were asked to choose between two products available that day–the eco-friendly paper and regular paper notebooks used in earlier experiments. Enforcement of choices and payment was the same as in earlier studies.

In the strong environmental-product set condition, participants were presented with 6 products (order counterbalanced), 4 of them environmentally friendly (i.e., pens and water bottles made of recycled plastic, reusable shopping bags and eco-friendly paper notebooks) and 2 non-environmentally friendly (i.e., a metal keychain and a regular paper notebook). In the weak-environmental-product set condition participants saw 6 products (order counterbalanced), 4 of them non-environmentally friendly (i.e., pens and water bottles made from virgin plastic, metal keychain and regular paper notebooks) and 2 environmentally friendly products (i.e., reusable shopping bag and an eco-friendly paper notebook). Next, all participants were asked to choose between the two notebooks. The product information in the catalog consisted of a photograph and five information bullet-point, with descriptions as in Experiment 1A. After participants made their decisions, they were asked to report on the same self-assessed emotions as in Experiments 1A and 1B plus a question about “a sense of being a good person” using the same scale. Participants answered the same final questions as in earlier studies.

*Results*. In the strong environmental-product set condition, 41% chose the eco-friendly notebook and 59% the regular notebook (*z* = 2.34, *p* = .01) while in the weak environmental set condition 56% chose the eco-friendly notebook and 44% chose the regular notebook (*z* = 1.49, *p* = .18). Treatment effects analysis (logit model with inverse probability weighting and regression adjustment) was used given the potential influence of various covariates on the choice of the notebook. We found a significant treatment effect (ATET = .26, *s*.*e*. = .10, *z* = 2.48, *p* = .01) in the weak-environmental product set condition, which means that on average, after controlling for specified covariates, the probability of choosing the eco-friendly notebook was 26% more in the weak environmental product set than in the strong set condition. Results suggest that the activation of personal norms was effective in driving people to responsibly choose pro-environmental products more often in weak environmental set conditions than in the constrained (strong environmental set) conditions.

We conducted standard linear regressions to determine main effects of choosing the eco-friendly notebook on emotions, controlling for attention to social comparison, pro-environmental attitudes, gender and income. We found that it reduces guilt (*B = -*.*91; t = -3*.*3; p =* .*00*), it reduces shame (*B = -1*.*05; t = -3*.*7; p =* .*00*), it does not affect regret (*B =* .*39; t =* .*94; t =* .*34*), it increases pride (*B = 1*.*05; t 2*.*06; p =* .*01*) and it increases feeling as a good person (*B = 1*.*17; t = 3*.*03; p =* .*00*). Based on these results, we again analyzed whether the influence of choosing the eco-friendly notebook on norm-related emotions is carried through the moderation of the choice architectures. Before conducting the moderation analysis, we checked for multicollinearity issues caused by the relationship between treatment and choice and found Variance Inflation Factors below 1.2. Using the PROCESS macro v.2.16 in SPSS, we estimated conditional linear regressions of the effect of choosing the responsible notebook on emotions moderated by the integral personal norm activating condition (strong or weak). Results of this analysis are reported in Table 5. For all emotions, people in the weak environmental product set displayed a stronger effect of choice on emotions than people in the strong product set. Furthermore, the moderation of the experimental condition was stronger for positive emotions than it was for negative ones. For guilt and shame, effects of choices under the strong set condition were less than in the weak set condition but still significant, whereas for regret, pride and feeling as a good person there was no significant effect of choice in the strong set condition. (See graphical displays of the moderation effects can be found in the S2 Fig).

**Table 5:** Conditional linear regressions of choice on emotions, moderated by product set type.

	Model 1: Guilt			Model 2: Shame			Model 3: Regret			Model 4: Pride			Model 5: Good person		
	Coeff. (s.e.)	t	p	Coeff. (s.e.)	t	p	Coeff.(s.e.)	t	p	Coeff.(s.e.)	t	p	Coeff. (s.e.)	t	p
Constant	.64 (.88)	.73	.47	.10 (.89)	.11	.91	1.67 (1.22)	1.37	.17	2.57 (1.25)	2.06	.04	3.45 (1.18)	2.92	.00
**Choice**	-.92 (.28)	-3.30	.00	-1.06 (.29)	-3.70	.00	-.51 (.39)	-1.32	.19	.99 (.40)	2.49	.01	1.27 (.38)	3.37	.00
Treatment dummy (1 = weak, 0 = strong)	-.19 (.27)	-.71	.48	-.18 (.27)	-.64	.53	-.05 (.37))	-.13	.90	.35 (.38)	.91	.36	-.32 (36)	-.88	.38
Choice*Product set	-.41 (.51)	-.81	.42	-.24 (.52)	-.45	.65	-1.58 (.71)	-2.22	.03	1.14 (.73)	1.56	.12	1.58 (.69)	2.30	.02
Attention to social comparison	.16 (.16)	.96	.34	.32 (.17)	1.89	.06	-.27 (.23)	-1.18	.24	-.18 (.23)	-.78	.44	-.15 (.22)	-.67	.51
Pro- Environmental predisposition	.18 (.12)	1.51	.14	.20 (.12)	1.68	.10	.20 (.16))	1.20	.23	.40 (.17)	2.39	.02	.28 (.16)	1.75	.08
Gender (1 = Male, 0 = Female)	-.22 (.26)	-.86	.39	-.31 (.26)	-1.17	.25	.36 (.369	.99	.32	.07 (.37)	.18	.86	-.20 (.35)	-.58	.56
Increase in R2 due to interaction	.01	F = .61	.42	.00	F = .21	.65	.06	F = 4.93	.03	.02	F = 2.44	.12	.05	F = 5.27	.02
Conditional effects of choice on emotions:															
Strong environmental set	-.72 (.36)	-1.99	.05	-.94 (.37)	-2.54	.01	.25 (.51)	.46	.63	.45 (.52)	.86	.39	.51 (.49)	1.04	.30
Weak environmental set	-1.14 (.39)	-2.88	.01	-1.18 (.40)	-2.93	.00	-1.33 (.55)	-2.43	.02	1.59 (.56)	2.82	.01	2.09 (.53)	3.94	.00
															
	R2 = .17	F = 2.47	.03	R2 = .22	F = 3.41	.01	R2 = .11	F = 1.43	.22	R2 = .28	F = 4.75	.00	R2 = .31	F = 5.29	.00
	N = 79			N = 79			N = 79			N = 79			N = 79		

Experiment 2 shows that putting the responsible product within a weak product increases the probability of pro-environmental product choices (26% more) and the resulting emotions suggest that activation of integral personal norms took place. In addition, choosing the pro-environmental notebook from the weak product set had a stronger impact on post-decision emotions, both negative and positive, than it did under the strong set for emotions as it was expected from the revised theories on norms and emotions. This effect was even more salient for positive emotions. As in experiment 1A, emotions were activated by choosing a product.

#### Experiment 3

*Methods*. We then focused on subjective social norms and tested another choice architecture intervention, one that makes social norms salient by making participants’ behavior subject to peer approval or disapproval; that is, we manipulated subjective social norms (i.e., injunctive norms) in ways similar to previous studies [18]. In Experiment 1A and1B, we used a social mechanism related to conformity to make descriptive social norms explicit, and we did not observe an effect on neither choice nor WTP. Since subjective social norms are enhanced by public exposure and enforced by social rewards and punishments [21, 77], this experiment tests whether knowing that one’s decision will become known to others modifies behavior.

*Participants*. For a priori power analysis, we evaluated the effect of treatment and other covariates on willingness to pay using a linear model as we did in Experiment 1B, and setting the same parameters. That is, to identify a small effect size (f^2^ = 0.1) with six covariates, alpha = 0.05 and power = 0.8 the resulting target sample size is 64 and critical t = 1.67. Participants were 80 undergraduate students (*M*_*age*_ = 19.2 (SD = 1.79; 50% female), all participants were included in the analysis.

*Procedure*. Participants were assigned to an experimental condition where their WTP for the eco-friendly notebook is made public. As a control, we used the same control group as in Experiment 1B, where WTP was examined without manipulation. At the start of the session, participants completed the same math problems as in earlier experiments and were paid in the same way for each correct answer. Next, they were told the same cover story (i.e., university web store) and asked to indicate their WTP for the pro-environmental notebook as in Experiment 1B. No descriptive norm of behavior was provided. Participants learned instead that they would have to report to the investigator how much they were willing to pay at the end of the experiment session, and that at the end of the day a list of names and WTP offers would be distributed to all study participants. Product information was the same as in the no-norm condition of Experiment 1B. Participants made their offers, reported on the same self-assessed emotional reactions as in earlier studies, and answered the same dispositional measures and demographic questions explained in the experiments overview section.

*Results*. Average WTP was $2.37, almost identical to the control group ($2.32) (t = .03; p = .94) suggesting that making offers public to the experimenter and other participants had no effect on WTP. Hence, no discernible influence on people choosing pro-environmental products was found from making subjective social-norms salient by the threat of offers being made public.

## General discussion

The findings in this research indicate that it is possible to use different choice architectures to nudge people to prefer pro-environmental products by making personal norms salient, but less so by making social norms salient. In addition, we find that such interventions activate norm-consistent feelings. We were able to match types of norms to choice architecture techniques, which resembled previously used interventions on social norms [6]. To the best of our knowledge, there is no antecedent in the choice architecture or norms literature that directly compares the efficacy of activating social versus personal norms for the same target behavior. Prior research has examined the interaction of subjective social norms and descriptive ones, and found that when goals are congruent, they additively influence behavior [78]. However, no comparison or interaction with personal norms have been described using the same target behavior (i.e., choosing the eco-friendly notebook). In particular, two types of choice architecture techniques were found to be most effective: Type 1, in which information about introjected personal norms is presented explicitly (pointing out correct behavior), and Type 2, in which decision structure was manipulated by varying product assortment contexts, which implicitly triggers integral personal norms.

The results also suggest that choice architecture interventions moderate the link between choice and norm-related feelings, even if preference for the pro-environmental notebook does not increase, as was the case with descriptive social norms. As expected, guilt and pride were more significantly linked to the choice made when introjected personal norms (low internalized) were activated, whereas shame and a sense of being a good person were more significantly linked to the choice made when social norms were activated. Also as expected, when integral personal norms were activated a wider emotional response was observed.

### Implications

Our results highlight the transformative potential of managed choice environments. We open a pathway for producers and policy makers to increase sustainable consumption without direct attempts to influence people’s dispositions. Moreover, finding that appeals to personal norms are more effective than appeals to social norms suggests that interventions may not need to depend on complex culture-influenced social mechanisms. Personal norms and social norms may be intertwined in complex ways as people may follow personal norms conditioned on empirical and descriptive social norms encountered in decisions contexts [79]. Nonetheless, the type of interventions we found effective in this work are based on existing personal norms that are internalized to different levels, and we activated internalization without placing the decision maker in a position to contrast personal and social norms. Our interventions tap into environmental concerns and beliefs that people hold, which research suggests are widely held [80]. We support the idea of continuing to emphasize the moral aspects of environmental issues [81], which complements research that shows the potential of moral framing in the promotion of prosocial behaviors [82, 83]. Moreover, our results inform the links between prosocial and PEB through appeals to personal norms and moral feelings.

Another noteworthy finding is that feelings were involved when choices were made but not when the WTP was elicited, even when social exposure was expected. It is established that social norms trigger both cognitive and affective brain processes [79, 84, 85]; processes in which social punishment for not complying plays a significant role. However, in a WTP task, there is no immediate behavioral reference to the acceptance or breaking of a norm from which specific emotions may ensue. In other words, there is no reference to the correct value to which WTP can be compared. It seems plausible, therefore, that the mechanisms involved in choice and willingness to pay responses differ in their elicitation and activation of emotions in as yet unexplored ways. The required valuation (i.e., WTP) in our experiments seems to be tapping only cognitive activity, whereas choice taps cognition and emotion. This result deserves further research in light of theories of preference elicitation techniques, but in closer relation to our goals, it highlights the importance of nudging people to start making pro-environmental choices that trigger emotional feedback mechanisms [70]. Our results indicate that using willingness to pay for socially or environmentally responsible products reinforces already-established concerns for sustainability, but it is not informative or useful in triggering actual behavior unless choice interventions that activate personal beliefs are implemented. Thus, by nudging people´s choices, through the activation of personal norms using choice architecture, the right feelings and meta-cognitions are elicited. We consider that feedback learning and body loop [86] mechanisms could be engaged, which may facilitate long term behavioral change with respect to sustainable consumption.

Our results imply that concern for the environment is likely part of people’s moral structure and constitutes a promising path to reducing the attitude-behavior gap in sustainable consumption. The link between choices and guilt, shame, regret, pride and sense of being a good person was highly significant, which is consistent with the observed enhancement of concern about the environment [80] and the moral nature of such concern [32]. Furthermore, the main effects of choice on positive feelings (pride) and affective meta-cognitions (sense of being a good person) were stronger than those of negative feelings (guilt, shame and regret). This opens the possibility of interventions that harness positive feelings, avoiding the problems of long-term effectiveness of negative feelings, such as negative spillovers [87]. Moreover, this also makes it possible to frame the promotion of PEB as a positive intervention [88] that targets people´s well-being instead of the avoidance of negative consequences. This may have impactful advances in the achievement of enduring sustainable lifestyles because people within positive interventions may associate PEB with positive emotions that engender expanded cognitive flexibility, creativity and perspective [89–91]. In particular, positive emotions that ensued from acting in accordance with personal norms may favor the adoption of PEB as part of a process of positive emotional regulation [88].

We also find close connections between Thøgersen’s [21] norm typology and studies on motivation towards environment-friendly behaviors conducted under the self-determination theory framework [25]. These studies have shown that self-determined motivation (i.e., more internalized and integrated motivations) is strongly correlated with environmentally responsible behaviors, in the short and in the long term [92–95]. Taken together, these studies support the conclusions of the present study that PEB may increase with norm internalization and integration (i.e., personal introjected norms and personal integrated norm) or the motivation to act in an environmentally responsible way.

Finally, although we reveal that nudging individuals can positively influence the adoption of PEB, researchers have called attention on possible unintended and even detrimental consequences of nudging [96, 97]. These unintended consequences go beyond merely an unsuccessful intervention, but may lead to actual backfiring effects that worsen outcomes [for a discussion, see 98, 99]. That is, there has been cases in which the intervention produced a detectable behavioral reaction contrary to what was intended and has subsequently worsen the welfare of the individual and the group. A few examples of such unexpected consequences of nudge-type interventions include an increase in energy consumption [100] and a decline in sustainable food choices [101] and support for environmental policies [102]. These findings are important because they highlight the potential risks of behavioral interventions affecting societal and economic outcomes at both individual and collective levels. Therefore, it is important to better understand why and when nudging can backfire and how to prevent this untainted consequences.

### Limitations

Setting up experimental studies that seek to activate norms requires simplifications of reality that generate caveats and limitations in the interpretations of results. One limitation of our design is that it does not allow for testing of the interaction between consistent personal and social norms, which opens an intriguing path of inquiry. The consistency or inconsistency of personal and social norms may have important effects in how people display norm-consistent behaviors. Regarding the complex dynamics of norms, another promising extension of our work is to test dynamic expressions of norms [36] for both personal and social norms.

Another limitation arises from the possible context dependency of the activation of personal norms. It was our goal to increase choices of pro-environmental products (by means of an environmentally friendly notebook), a context which already seems to trigger high concern and strong beliefs as revealed by multiple surveys applied around the world. In other contexts, where the presence of references for personal norms are less widespread, the relative strength of personal versus social norms may vary.

Our work explicitly controlled for individual dispositions towards pro-environmental values and attention to social comparison as alternate influencers of choice behavior, which may have confounding effects on choices. There may be other dispositional factors, not controlled in our experiments, that may also influence consumption and may affect the effectiveness of personal and social norm interventions to promote pro-environmental choices in various ways. Some individual level dispositional factors are materialism [103] and perceived consumer effectiveness [104], which should be considered in future research. In any case, there is no clear conceptual basis to hypothesize how those factors would neutralize or supersede the social and personal norms effects found here.

Finally, another set of limitations ensue from our emotional measure and theory choices. As explained, we used a self-reported method to measure emotions, which requires some reflection by participants on their feelings and the attribution of such feelings to specific choices. Other emotion capturing techniques and theories, such as integral versus incidental emotions, may prove useful in replicating, confirming, or setting boundary conditions to our results.

### Final remarks

The path to achieve sustainable consumption and to harness its full benefit to the environment is still long and steep. Behavior change strategies may be directed to multiple stages along the individual decision-making processes. Our results offer avenues to reduce the attitude-behavior gap that uses individual level beliefs and personal norms, thereby circumventing the idiosyncratic complexities of dynamic social norms. Current evidence shows that concern for the environment and climate change are already widespread [80], which makes us optimistic about consumers’ internalization of personal norms, but such norms need to be activated in order to shape behaviors. According to our results, choice architecture offers a fast and straightforward way to trigger PEB, while potentially engaging long term learning mechanisms based on mostly positive feelings.

## Supporting information

S1 Fig Interactive effects of choice and treatment in Experiment 1A. (TIF)

S2 Fig Interactive effects of choice and treatment in Experiment 2. (TIF)
