# Supplementary figures and images for: Norm-focused nudges influence pro-environmental choices and moderate post-choice emotional responses
Source: PLoS One. 2021 Mar 1;16(3):e0247519. doi: 10.1371/journal.pone.0247519 (PMC7920343; doi:10.1371/journal.pone.0247519)

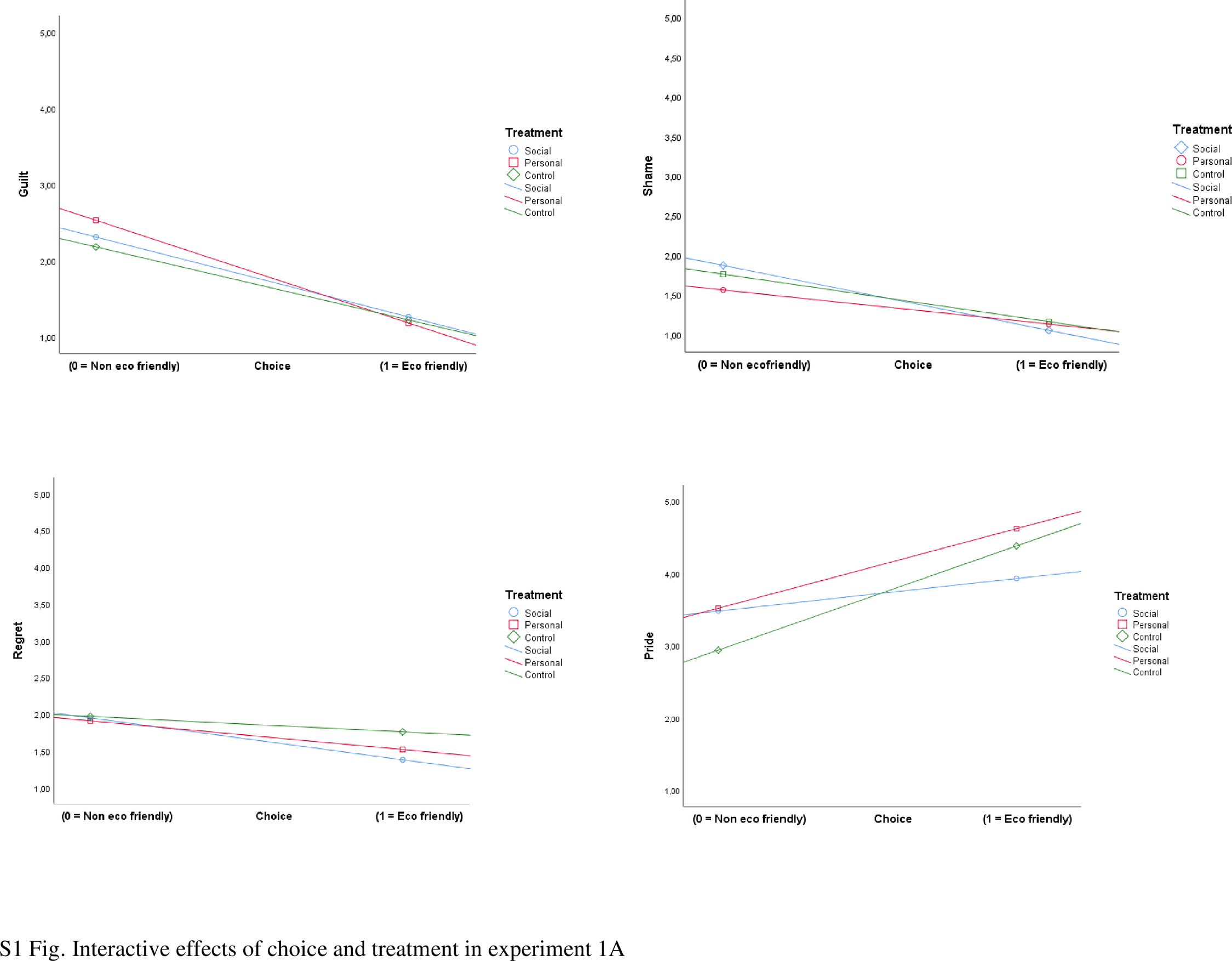

Supplement: S1 Fig — (TIF) [file pone.0247519.s001.tif]

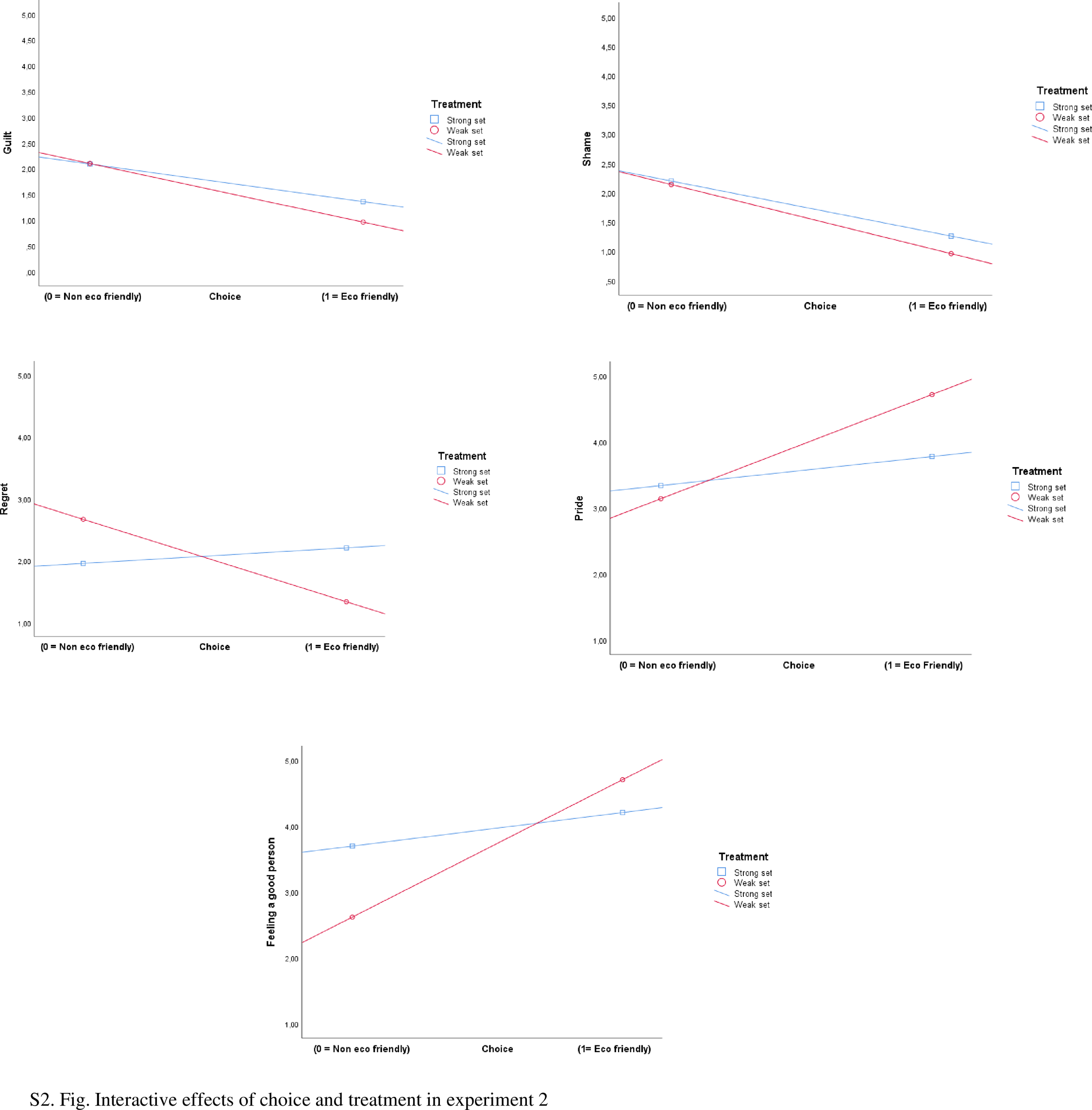

Supplement: S2 Fig — (TIF) [file pone.0247519.s002.tif]
